# Less invasive surfactant administration versus endotracheal surfactant instillation followed by limited peak pressure ventilation in preterm infants with respiratory distress syndrome in China: study protocol for a randomized controlled trial

**DOI:** 10.1186/s13063-020-04390-3

**Published:** 2020-06-11

**Authors:** Jiajun Zhu, Yingying Bao, Lizhong Du, Huafei Huang, Qin lv, Yejun Jiang, Yuxuan Dai, Zhijun Chen, Jingyun Shi, Yongyan Shi, Chuangzhong Yang, Hua Mei, Hong Jiang, Yanhui Sun, Xuemei Sun

**Affiliations:** 1 Women’s Hospital, Zhejiang University, School of Medicine, Hangzhou, 310006 China; 2 The Children’s Hospital, Zhejiang University, School of Medicine, Hangzhou, 310052 China; 3 Jiaxing Maternity and Child Health Care Hospital, Jiaxing, 314051 China; 4 Ningbo Maternal and Children Hospital, Ningbo, 315012 China; 5 Shao Xing Maternity and Child Health Care Hospital, Shaoxing, 312000 China; 6 Central Hospital of Jinhua, Jinghua, 321000 China; 7 Boai Hospital of Zhongshan, Zhongshan, 528400 China; 8 Gansu Provincial Maternity and Child-Care Hospital, Lanzhou, 730050 China; 9 Shengjing Hospital of China Medical University, Shenyang, 110004 China; 10 Affiliated Shenzhen Maternity& Child Healthcare Hospital, Southern Medical University, Shenzhen, 518028 China; 11 Affiliated Hospital of Inner Mongolia Medical University, Hohhot, 010050 China; 12 Yan’an University Affiliated Hospital, Yan’an, 716000 China; 13 Chongqing Health Center for Women and Children, Chongqing, 400021 China; 14 Linyi People’s Hospital, Linyi, 276003 China

**Keywords:** Less invasive surfactant administration, Low peak pressure ventilation, Respiratory distress syndrome, Preterm infant

## Abstract

**Background:**

Less invasive surfactant administration (LISA) is a way of giving surfactant without endotracheal intubation and has shown to be promising in reducing the incidence of bronchopulmonary dysplasia (BPD) in preterm infants. However, the mechanism underlying its beneficial effect and variations in the technique of administration may prevent its widespread use. This trial aims to evaluate the effects of two methods of surfactant administration, LISA or endotracheal surfactant administration followed by low peak pressure (LPPSA) ventilation, in preterm infants with respiratory distress syndrome (RDS).

**Methods:**

The **L**ISA **O**r **L**ow **P**eak **P**ressure trial is to be conducted in 14 tertiary neonatal intensive care units in China. A total of 600 preterm infants born with gestational age between 25^0/7^ and 31^6/7^ weeks and with a primary diagnosis of RDS will be involved in the study. Infants will be randomized to the LISA or LPPSA group when surfactant therapy is indicated. Primary outcomes include mortality, severity of bronchopulmonary dysplasia at 36 weeks of postmenstrual age (PMA), and mechanical ventilation (MV) in the first 72 h of life. Secondary outcomes include the days of MV, duration of all sorts of non-invasive respiratory support, fraction of inspired oxygen, oxygen saturation before and after surfactant administration, and time required to perform the procedure for surfactant administration. The incidence of comorbidities, including retinopathy of prematurity (ROP), necrotizing enterocolitis (NEC), intraventricular hemorrhage (IVH), hemodynamically significant patent ductus arteriosus (hsPDA), pneumothorax, and massive pulmonary hemorrhage within 48 h of surfactant administration, and the failure rates of each technique will be determined.

**Discussion:**

Data from recent systematic review and meta-analysis have suggested a possible improvement in outcomes of preterm infants with RDS by the LISA technique. However, robust evidence is lacking. Why LISA plays a potential role in reducing respiratory morbidity, mainly BPD in preterm infants, remains unclear. The possible explanations are the active and uninterrupted delivery of continuous positive airway pressure during the LISA procedure and the avoidance of complications caused by intubation and relatively high pressure/volume ventilation following surfactant administration. We hypothesized that LISA’s effectiveness lies mainly in avoiding relatively high-pressure positive ventilation immediately following surfactant administration. Thus, this multicenter randomized controlled trial will focus on issues of endotracheal intubation and the pressure/volume used during conventional surfactant administration. The effectiveness, safety and comorbidities of preterm infants following LISA or LPPSA will be evaluated.

**Trial registration:**

Chinese Clinical Trial Registry: ChiCTR1900020970. Registered on 23 January 2019.

## Background

In the last several years, dramatic improvements have been made in the management of respiratory distress syndrome (RDS) in preterm infants, including early establishment of FRC by noninvasive respiratory support, early rescue instead of prophylactic surfactant use, and curtailed application of mechanical ventilation (MV). All these changes have greatly improved the outcome for babies with RDS [1, 2].

Less invasive surfactant administration (LISA), as an innovative mode of surfactant delivery, is getting increasing attention in the management of preterm infants with RDS. This technique is characterized by the use of a thin catheter to deliver surfactant instead of an endotracheal tube and by keeping continuous positive airway pressure (CPAP) support during the course of administration [3]. A recent systemic review and meta-analysis in preterm infants with RDS indicated that surfactant administration via the LISA method decreased the combination rate of mortality or bronchopulmonary dysplasia (BPD) and reduced need for MV during the first 72 h of life [4]. However, the implementation of the technique requires an experienced clinician and consistence when performing the procedure. Moreover, the mechanisms underlying the beneficial effect of LISA over conventional methods are not fully understood, and further research is warranted [5]. While the intubation-surfactant-extubation (INSURE) technique as a conventional method has been widely used, evidence on superiority, efficacy, and safety of the LISA method over conventional surfactant administration are needed.

To provide more evidences and elucidate the potential protective mechanisms of the LISA method, we therefore conduct a multicenter, prospective, randomized controlled trial in China. This trial aims to compare the effects of the LISA method with those of endotracheal surfactant administration immediately followed by positive pressure ventilation with a designated lower peak pressure than it does in INSURE (LPPSA) in preterm infants with RDS and indicated for surfactant administration. Our hypothesis is that the effects of designated low positive pressure ventilation use following conventional surfactant administration (LPPSA) are not inferior to LISA in the treatment of preterm infants with RDS.

## Methods/design

### Study aim

This trial aims to compare the mortality and incidence of BPD at 36 weeks of PMA and the MV requirement during the first 72 h of life between LISA and LPPSA methods.

### Setting

**Fig. 1:**
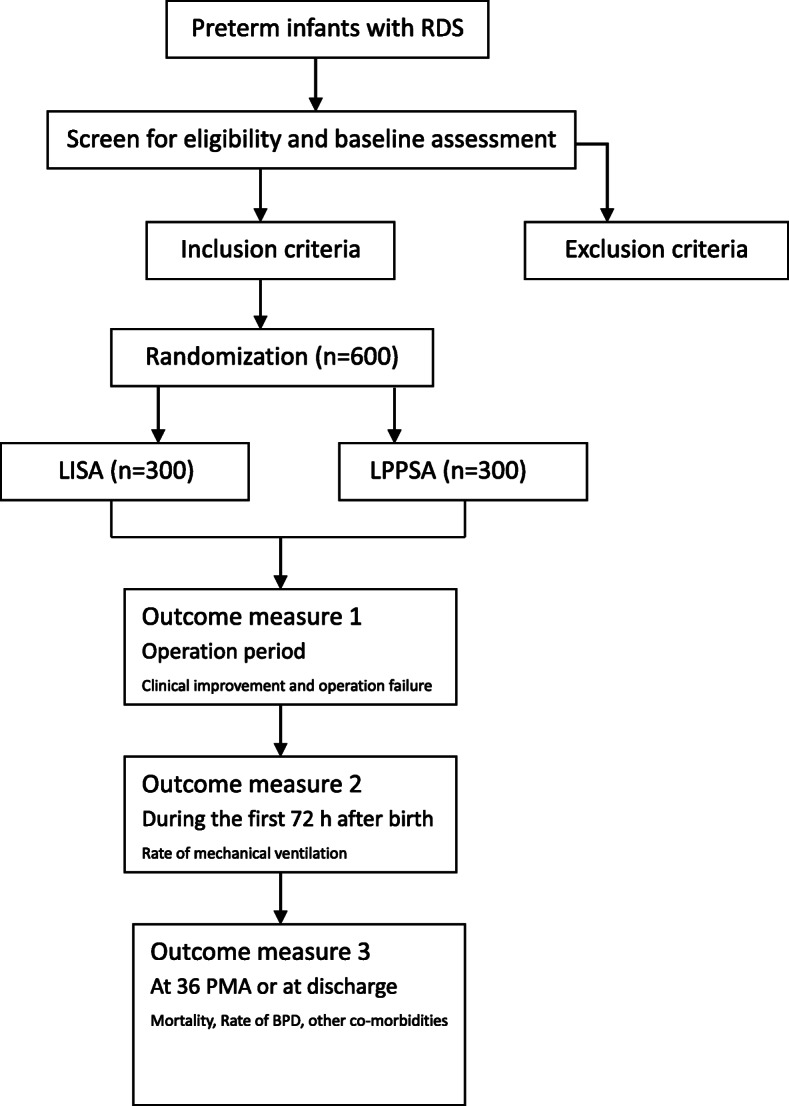
Flow diagram of the trial protocol. *LPPSA* surfactant administration followed by low peak pressure ventilation, *PMA* postmenstrual age, *LISA* less invasive surfactant administration

The **L**ISA **O**r **L**ow **P**eak **P**ressure trial, a multicenter randomized prospective trial, will be conducted in 14 tertiary neonatal intensive care units (NICUs) in China from January 2019 to December 2020. The trial has been approved by the ethics committee of the Women’s Hospital School of Medicine, Zhejiang University. This protocol has been registered in the Chinese Clinical Trial Registry (ChiCTR1900020970). The diagram of the study protocol is presented in Fig. 1.

### Grouping

Infants will be divided into two groups, namely, the LISA group or LPPSA group, according to the method of surfactant administration. Infants in the LISA group will receive surfactants by using a thin catheter (LISA catheter® designed by Shuanghe Pharmaceutical, Beijing). Infants will be on nasal continuous positive airway pressure (NCPAP) support during the course of surfactant administration. Infants in the LPPSA group will be given surfactant by traditional intubation and MV support with a T-piece or ventilator immediately following surfactant administration as done in INSURE but with a peak inspiratory pressure at < 15 cmH_2_O (12–15 cmH_2_O), positive end-expiratory pressure (PEEP) at 6–8 cmH_2_O, and respiratory rate at 40–60 breaths per minute.

### Inclusion criteria

Infants will be included if they meet all of the following criteria:

1. Born between 25^0/7^ and 31^6/7^ weeks of gestation with birth weight between 600 g and 1500 g.
2. Exhibit vigorous spontaneous breathing and can be stabilized by noninvasive respiratory support
3. With PEEP at 6–8 cmH_2_O and in need of fraction of inspired oxygen (FiO_2_) ≧0.3
4. Less than 6 h of age
5. Informed parental consent has been obtained

### Exclusion criteria

Infants will be excluded if they meet any of the following:

1. Requiring MV or intubation in the delivery room
2. Presence of major congenital malformation or chromosomal abnormality or inherited disorders of metabolism
3. Have neuromuscular diseases affecting respiratory function
4. Presence of congenital pneumonia or pulmonary hypoplasia

### Randomization

A randomization sequence with 600 numbers (odd to even numbers in a 1:1 ratio) will be generated by a computerized random number generator and placed in sealed opaque envelopes. Infants with odd and even numbers will be assigned to the LISA and LPPSA groups, respectively. Twins or multiple birth infants will be randomized separately. Every eligible infant will be allocated to each group according to a randomized sequence within 30 min by an independent person (a nurse in Women’s Hospital School of Medicine, Zhejiang University).

### Blinding

Operators and care providers will not be blinded, and the outcome assessors and data analysts will be blinded to the intervention. Decisions regarding ongoing treatments will be made according to every unit’s guidelines and clinical practice. The authors had two meetings before the study to obtain consensus for neonatal care.

### Sample size calculation

The proposed sample size is calculated according to the incidence of BPD. According to previous reports and the average data from every unit involved in the trials [6, 7], approximately 60% of infants born at 25^+ 1^ to 27^+ 6^ gestational weeks and approximately 15% among those born at 28^+ 1^–31^+ 6^ gestational weeks have developed BPD during the past 5 years. The presumed incidence of BPD will be approximately 25% in the pooled population of our trial. Considering an alpha error rate of 0.05 and a power of 0.8 to detect an expected 20% reduction in the incidence of BPD, 258 infants will be enrolled in each group. To consider factors such as data incompleteness, failure to follow up, or early dropout, we plan to recruit 300 preterm neonates for each group.

### Intervention

Positive pressure with a T-piece system (Neopuff Infant Resuscitation®, Fisher and Paykel, Auckland, New Zealand) by a suitable mask will be applied to stabilize the infant after birth. Doctors in each participating center should be experienced using both methods. One must have experience with at least 10 cases implementing each technology.

#### LISA method

Oral sucrose will be encouraged during the whole procedure, and caffeine or any sedating drugs before operation will not be allowed in our trial. A specified thin catheter (1.67 mm in diameter and 150 mm long, designed by Shuanghe Pharmaceutical, Beijing) is inserted beyond the vocal cords to the required depth (25–27 weeks at 1 cm, 28–31 weeks at 1.5 cm) under direct vision by laryngoscopy. Surfactant will be administered through the thin catheter within 2–3 min. During the procedure, the infant will be on NCPAP with a PEEP of 6–8 cmH_2_O and will receive tactile stimulation by the operator or nurse, who will rub the back or flick the sole, to maintain spontaneous breathing during the whole surfactant administration. The catheter will be immediately withdrawn after surfactant administration. If the infant develops apnea, bradycardia, or desaturation, surfactant administration will be stopped immediately. If the condition does not improve in 30 s, a positive pressure ventilation will be given to recover the infants, as previously described [1, 6], and the case will be recorded as a failure of LISA.

#### LPPSA method

The same principle of premedication will be applied. Surfactant will be administered through an endotracheal tube under direct vision by laryngoscopy. Surfactant will be continuously administered through an endotracheal tube with mechanical ventilator or T-piece resuscitator. The initial setting will be as follows: PEEP of 6–8 cmH_2_O, peak inspiratory pressure of 12–15 cmH_2_O, and respiratory rate of 40 breaths per minute. If the infant develops apnea, bradycardia, or desaturation, surfactant administration will be stopped immediately. If the condition does not improve in 30 s, then conventional peak pressure ventilation (18–20 cmH_2_O or more), will be given, and the case will be considered as a failure case of LPPSA.

#### Surfactant dosage

Use of porcine surfactant (Curosurf®, Chiesi, Italy) or bovine surfactant (Calsurf®, Shuanghe, Pharmaceutical, Beijing) will be allowed in this trial. Based on literature concerned on the efficacy of different doses of surfactant and also from our experience, 200 mg/kg of porcine surfactant or 100 mg/kg of bovine surfactant will be used in the study. For infant with RDS as the primary cause, a second or third dose of 100 mg/kg (porcine) or 50 mg/kg (bovine) may be used if the infant’s condition is deteriorated and RDS is still the primary consideration.

#### CPAP and nasal intermittent positive-pressure ventilation (NIPPV)

Both CPAP and NIPPV may reduce the morbidity of BPD in preterm infants when they are used as primary respiratory support. However, no consensus exists on the optimal setting value. In the trial, we use NCPAP as the primary noninvasive respiratory support during and after surfactant administration. The initial setting of PEEP will be at least 6 cmH_2_O (6–8 cmH_2_O). Both constant and variant flow NCPAP equipment are permitted to use in the trial based on the individual requirement. The proximal end of the gastric tube will be kept open to avoid abdominal distension, and soothing care such as use of pacifier or chin fixer can be used to achieve the expected pressure.

#### MV indication

The infant will receive MV therapy if any of the following condition occurs: (1) FiO_2_ ≧ 0.45, PEEP > 8 cmH_2_O, SPO_2_ < 90%, and the condition lasts > 15 min; (2) frequent or severe apnea even after the caffeine therapy. In the trial, ≥ four episodes of apnea requiring vigorous stimulation in 6 h is defined as frequent apnea, and severe apnea defined as ≥ 2 episodes requiring positive pressure ventilation in 6 h; (3) persistent (confirmed by two arterial blood gas samples at least 30 min apart) respiratory acidosis with pH < 7.20 and PCO_2_ > 65 mmHg; (4) critical circumstances requiring intubation such as cardiac arrest caused by severe metabolic acidosis [8]. These criteria only apply to infants aged ≤ 1 week, and will be used only for the first time after birth to judge whether MV management should be conducted instead of noninvasive ventilation.

#### Indication for the weaning from MV

The infant will be weaned from MV if all the following criteria are achieved. All preterm infants in this trial will receive caffeine therapy at least 24 h before weaning. Caffeine therapy with a loading dose of 20 mg/kg and then a maintenance dose of 5–10 mg/kg is encouraged in the trial. These criteria are as follows:

1. The arterial blood gas should be maintained in a targeted range (pH ≧ 7.20, PaO_2_ ≧ 50 mmHg, PaCO_2_ ≦ 60 mmHg) with a low positive pressure (mean arterial pressure (MAP) ≦ 7 cmH_2_O, FiO_2_ ≦ 0.3).
2. Stable spontaneous breathing should be present.

#### Indication for noninvasive support weaning

The infant will be weaned from NCPAP or NIPPV, if it has been 24 h since all of the following criteria will be achieved:

1. MAP/PEEP ≦ 3–5 cmH_2_O
2. FiO_2_ ≦ 0.25
3. No apnea and bradycardia that requires stimulation

#### Termination of the study

1. Death in hospitalization
2. Early dropout on account of parents’ decision

### Outcomes

#### Primary outcomes

The primary outcomes will be as follows:

1. Mortality, co-morbidity, and severity of BPD at 36 weeks of corrected gestational age.
2. MV requirement in the first 72 h of life.

#### Secondary outcomes

The secondary outcomes will be as follows:

1. Need for and duration of MV (days) during of hospitalization
2. Duration of noninvasive respiratory support (days)
3. Duration of oxygen need.
4. Heart rate and oxygen saturation before and after surfactant administration in every 30 s for 10 min.
5. Incidence of pneumothorax and massive pulmonary hemorrhage within 48 h of surfactant administration
6. Severe neonatal diseases including and hemodynamically significant patent ductus arteriosus (hsPDA) that needs medical or surgical intervention
7. Duration of hospitalization
8. Failure rate of operation (including LISA or LPPSA technique)
9. Time required to perform the procedure of surfactant administration.

### Other data

Other infant information that will be collected is as follows: gestational age, birth weight, sex, delivery mode, Apgar score at 1 and 5 min, antenatal steroid use, prolonged premature rupture of membrane (> 18 h), intraventricular hemorrhage (IVH, grade 3–4), stage II-III necrotizing enterocolitis (NEC), retinopathy of prematurity (ROP, ≧ III stage), and postnatal steroid use.

### Definitions

#### BPD

BPD is defined as postnatal treatment with oxygen > 21% for at least 28 days, plus oxygen requirement at 36 weeks PMA [9]. According to our inclusive criteria, the time point of the assessment is 36 weeks PMA or discharge to home, whichever comes first, and the grading criteria are as follows: (1) mild, breathing room air; (2) moderate, need for < 30% oxygen; and (3) severe, need for ≥30% oxygen and/or positive pressure support [9]. If the infant dies in the early days of life because of lethal BPD, a special case will be recorded separately according to revised definition of BPD [10].

#### NEC (proven and advanced)

Proven NEC (stage II) encompasses the signs of stage I plus the absence of bowel sounds with or without abdominal tenderness. Abdominal tenderness is present, and some infants have cellulitis of the abdominal wall or a mass in the right lower quadrant. Infants with stage IIA are mildly ill, whereas those with stage IIB NEC are moderately ill and have mild metabolic acidosis and thrombocytopenia. Findings on abdominal imaging include intestinal dilation, ileus, ascites, and pneumatosis intestinalis, which is the defining feature of stage II.

Advanced NEC (stage III) is the most severe form. In stage IIIA, the bowel is intact, whereas stage IIIB is characterized by bowel perforation visualized as a pneumoperitoneum on the abdominal radiograph. Infants with advanced NEC are critically ill. In addition to the signs shown in the less severe stages, they typically have hypotension, bradycardia, severe apnea, and signs of peritonitis (abdominal distention and marked tenderness). Laboratory signs include a combined respiratory and metabolic acidosis, neutropenia, and disseminated intravascular coagulation [11].

#### IVH (grades III and IV)

IVH with grade III is defined as IVH involving more than 50% of the ventricular area; lateral ventricles are usually distended. IVH with grade IV is characterized by hemorrhagic infarction in the periventricular white matter ipsilateral to IVH [12].

#### Severe ROP

Severe ROP, defined as ROP needing surgical intervention, including intravitreal injection, laser therapy, and cryotherapy, will be recorded in our study [13].

#### Massive pulmonary hemorrhage

This condition is characterized by the presence of hemorrhagic fluid from the endotracheal tube, accompanied by a sudden respiratory distress and deterioration of clinical condition (increased parameters or MV therapy is required with 1 h of the occurrence of blood fluid). X-ray imaging suggests involvement of more than two lung lobes [14].

#### Pulmonary air leak in the newborn

This condition is characterized by air leak from the lung identified by X-ray imaging, including pneumothorax, pneumomediastinum, pulmonary interstitial emphysema, and pneumopericardium.

#### Adverse events

Serious adverse events (SAEs) include death, prolonged hospitalization, and persistent disability, which are expected to be closely related to surfactant administration in the opinion of local medical investigators. SAEs will be reported within 24 h to the local Ethics Committee and Data and Safety Monitoring Committee (DSMC), and will be reported within 3 working days to coordinating centers. Any recommendations will be disseminated to local investigators. Adverse events are characterized by operation failure as described above or any clinical deterioration, including BPD, proven or advanced NEC, IVH (grades III and IV), severe ROP, massive pulmonary hemorrhage and pulmonary air leak, which are related to surfactant administration. AEs will be reported to the coordinating centers every month and will be closely monitored by the DSMC.

### Data collection

All data will be collected from patient records. Data will be entered by the doctor who participated in the study of the individual NICU on a web-based electronic case record form and a written case report form. Access to the form will be protected by password, and infants may be identified by number only. A full-time coordinator will be responsible for monitoring the progress of the study and collecting feedback information from each unit. Data will be collected at the following schedule (Table 1).

**Table 1:** Timeline of the study and clinical data collection

TIMEPOINT	Study Period				
	Enrollment	Allocation	Post-allocation		
	Birth to 0	0	Surfactant administration (-10min-+10min)	72 hours after birth	At 36 weeks PMA or at discharge
**Enrolment:**					
**Eligibility screen**	X				
**Informed consent**	X				
**Allocation**		X			
**Intervention**					
LISA			X	X	X
LPPSA			X	X	X
**Assessments**					
**Primary outcomes** 1.Need for ventilation during the first 72 hours				X	
2. Mortality 3. BPD					X
**Secondary outcomes** Need for and the duration of MV (days), duration (days) of non-invasive respiratory support, severe co-morbidities, pneumothorax, massive pulmonary hemorrhage within 48 h of surfactant administration, hemodynamically significant patent ductus arteriosus that needs medical or surgical intervention, Duration of hospitalization, Failure rate of LISA or LPPSA			X	X	X

*LPPSA* surfactant administration followed by low peak pressure, *LISA* less invasive surfactant administration

### Data monitoring committee

An independent Data Monitoring Committee (DMC) belonging to the Central Ethics Committee of Women’s Hospital School of Medicine, Zhejiang University, has been established for the trial. This committee will perform interim data analysis, investigate compliance with the trial, and monitor adverse events.

### Statistical methods

All data will be analyzed using SPSS version 20.0. To compare primary and secondary outcomes between the two groups, Student’s t-test (continuous variables) and the chi-squared test (categorical variables) will be used. The two-level hierarchical linear regression model or the logistic regression model, whichever is appropriate, will be applied for the comparison of the outcomes between the two groups, accounting for the infants’ characteristics, such as gestational age, birthweight, sex, appropriateness for gestational age, delivery mode, and pregnancy complications, and center variables, such as number of beds in the NICU and the number of infants with gestational age < 32 weeks. *P* < 0.05 will be regarded as statistically significant.

## Discussion

In recent years, the LISA method has been accepted by increasing number of doctors. However, the potential benefit of LISA is still not clear. Moreover, an increased incidence of spontaneous intestinal perforation in extremely premature infants has been reported [15]. A meta-analysis showed that LISA showed effects on reducing the rate of MV during the first 72 h and the combination of BPD mortality and morbidity based on previous trials, which compared the effects of LISA with conventional surfactant technique (including intubation and ventilation by bagging or ventilator). We found that previous studies focused more on the procedure of performing LISA but less on the clarity of the potential mechanism [16, 17]. In fact, pulmonary compliance will change during the period of surfactant administration, and the peak pressure of ventilation barely changed spontaneously, even with use of a more advanced ventilator. A preliminary idea is that the whole procedure of surfactant administration without ventilation may contribute most to the advantages of LISA [18]. Thus, if correct, we would have further comprehension of LISA, and further knowledge would encourage more efforts on applying optimal peak pressure to surfactant delivery instead of trying to require all doctors to learn the method of LISA in China.

The delivery of surfactant as soon as possible has been known to help the distribution of the surfactant [19] In a previous study, the LISA procedure may took 0.5–5 min (average 2-3 min), which is longer than that of conventional method. Desaturation and apnea are the main complications of LISA [20, 21]. This situation is seldom seen in conventional surfactant administration. The contradiction of “time and effects” between LISA and conventional method may suggest some potential benefits of LISA. In this trial, the time required to perform the procedure would also be evaluated, because of the variable data of previous studies.We hope it will give us more information of the reason and a relatively standard operation of LISA can be set.

To our knowledge, this trial will be the first multicenter randomized controlled trial to evaluate the potential benefits of LISA and will give a more objective evaluation of LISA.

### Trial status

The protocol version is 1.0, and the issue date is 18 December 2018. Recruitment of participants started in January 2019 and is ongoing. Recruitment of participants is expected to end no later than December 2020.

## Supplementary information

**Additional file 1.**

## Data Availability

Not applicable.

## Abbreviations

BPD: Bronchopulmonary dysplasia
FiO_2_: Fraction of inspired oxygen
ICH: Intraventricular hemorrhage
MAP: Mean arterial pressure
MV: Mechanical ventilation
NCPAP: Nasal continuous positive airway pressure
NEC: Necrotizing enterocolitis
NICU: Neonatal intensive care unit
PEEP: Positive end-expiratory pressure
RDS: Respiratory distress syndrome
ROP: Retinopathy of prematurity
LISA: Less invasive surfactant administration
LPPSA: Low peak pressure surfactant administration
