# Supplementary material for: Less invasive surfactant administration versus endotracheal surfactant instillation followed by limited peak pressure ventilation in preterm infants with respiratory distress syndrome in China: study protocol for a randomized controlled trial
Source: Trials. 2020 Jun 11;21:516. doi: 10.1186/s13063-020-04390-3 (PMC7289227; doi:10.1186/s13063-020-04390-3)
Supplement: Supplementary file 1 — Additional file 1. [file 13063_2020_4390_MOESM1_ESM.zip › renamed_8e1fdR2.pdf]

研究中心编号<br>□ □	受试者姓名缩写<br>□ □ □ □	受试者编号<br>□ □ □ □

改良微创肺表面活性物质运用与低吸气峰压支持肺表面活性物质滴入技术在极早早产儿呼吸窘迫综合征中应用比较的多中心临床随机对照研究

病 例 报 告 表

（Case Report Form）

入组情况：□ LISA 组　　□对照组

研究结束情况：□完成　　□退出

研究医师：________________________________

研究开始日期：________年_______月_______日

研究结束日期：________年_______月_______日

研究负责单位：浙江大学医学院附属妇产科医院

填写说明

<u>在正式填表前，请认真阅读下列填写说明</u>

病例报告表填写说明

1. 筛选合格者填写正式病例报告表。
2. 病例报告表应用圆珠笔填写
3. 病例填写务必准确、清晰、不得随意涂改、错误之处纠正时用横线居中划出，并签署修改者姓名拼音缩写及修改时间。　　举例：~~2.5%~~　　25% ZJJ-2017-6-20
4. 患者姓名缩写四格需填满，两字姓名填写两字拼音前两个字母，三字姓名填写三字拼音首字母及第三字拼音第二个字母，四字姓名填写四字拼音首字母，五字姓名及以上填写前四字拼音首字母。

举例：张红	Z	H	H	O
张小红	Z	X	H	O
张小小红	Z	X	X	H
张小小红红	Z	X	X	H

5. 所有选择项目的 □ 内用√标注。如：√。表格中所有栏目均应填写相应的文字和数字，不得留空。
6. 因故未查或漏查，请填写“ND”；具体用药剂量和时间不明，请填写“NK”；不适用请填写“NA”。
7. 如实填写不良事件记录表。记录不良事件的发生时间，严重程度，持续时间，治疗措施和转归。如有严重不良事件

发生（包括临床研究过程中发生需要转院治疗，延迟住院时间，伤残，影响功能、危及生命或死亡等事件），必须立即通知研究负责单位浙江大学医学院附属妇产科医院临床研究机构伦理委员会。

研究中心编号<br>□ □	受试者姓名缩写<br>□□□□	受试者编号<br>□□□□	筛选期记录

筛选期记录

基本信息
床号：□ □ 住院号：____________<br>性别： 男 □ 女 出生时间：201 □年 □□ 月 □□ 日□□时 □□ 分<br>孕周：□□周 天 日龄：□小时 □□ 分<br>出生体重：□□□□克 身长□□ cm 头围 □□cm<br>签署知情同意书日期：201 □ 年 □□月 □□ 日□□时 □□分<br>并发症： 无 有 如有请填写____________
受试者是否符合入选标准

1. 胎龄 25^{+0}周～31^{+6}周	是 □	否 □
2. 出生体重在<1500g	是 □	否 □
3.生后 6 小时内	是 □	否 □
4.无创通气支持，PEEP 5-7cmH_2O，自主呼吸活跃	是 □	否 □
5.吸入氧浓度>0.3	是 □	否 □
6.同意外源性肺表面活性物质治疗	是 □	否 □
7.家属已签署知情同意书	是 □	否 □

如果以上任何一项回答是“否”，则受试者不能进入本次临床研究。

研究中心编号<br>□ □	受试者姓名缩写<br>□□□□	受试者编号<br>□□□□	筛选期记录

筛选期记录

受试者是否符合排除标准		
1.已接受气管插管治疗或存在气管插管治疗指证	是 □	否 □
2. 明显的先天畸形或染色体疾病	是 □	否 □
3. 存在影响呼吸功能神经肌肉疾病	是 □	否 □
4. 呼吸道结构异常	是 □	否 □
5. 先天性肺发育不良	是 □	否 □
6.呼吸困难严重，研究者认为不能耐受 LISA 操作	是 □	否 □

如果以上任何一项回答为“是”，则受试者不能参加本次临床研究

研究医师（签名）：________________

日期：201□ 年 □□月 □□ 日□□时 □□分

复核人（签名）：__________________

日期：201□ 年 □□月 □□ 日□□时 □□分

研究中心编号<br>□ □	受试者姓名缩写<br>□□□□	受试者编号<br>□□□□	观察期记录

观察期记录（用药前 30 分钟）

生命体征：

体温：□□.□ ℃　　**心率：**□□□次/分

呼吸：□□ 次/分　　**血压：**□□/□□ mmHg

经皮脉搏氧饱和度：□□ %

呼吸支持方式：

□NCPAP □BiPAP □NIPPV □sNIPPV □ nHFO

呼吸支持参数：

FiO2：□□%；　　PEEP □□ cmH_2O

（选填） PIP: □□cmH_2O **频率:** □□次/分或□□ HZ

吸气时间□.□ **秒　振幅：**□□ cmH2O

研究医师（签名）：____________________

日期：201□ 年 □□月 □□ 日□□时 □□分

复核人（签名）：____________________

日期：201□ 年 □□月 □□ 日□□时 □□分

研究中心编号<br>□ □	受试者姓名缩写<br>□□□□	受试者编号<br>□□□□	观察期记录

观察期记录（用药前后 5 分钟）

用药前 5 分钟：

生命体征：

体温： □.□ ℃ **心率：**□□□ 次/分

呼吸： □□次/分 **血压：**□□/□□ mmHg

经皮脉搏氧饱和度：□□ %

呼吸支持方式：

□NCPAP □BiPAP □NIPPV □sNIPPV □nHFO

呼吸支持参数：

FiO2：□□%； PEEP □□ cmH_2O

（选填） PIP: □□cmH_2O **频率：** □□次/分或□□ HZ

吸气时间 □.□ **秒** **振幅：**□□ cmH2O

研究医师（签名）：________________

日期：201□ 年 □□月 □□ 日□□时 □□分

复核人（签名）：________________

日期：201□ 年 □□月 □□ 日□□时 □□分

研究中心编号<br>□ □	受试者姓名缩写<br>□□□□	受试者编号<br>□□□□	观察期记录

观察期记录 用药开始时间201□年□□月□□日□□时□□分

用药结束时间 201□年□□月□□日□□时□□分

开始用药0秒：

心率：□□□次/分　　呼吸：□□次/分□□

吸入氧浓度：□□ %　　经皮脉搏氧饱和度：□□ %

用药30秒

心率：□□□次/分　　呼吸：□□ 次/分

吸入氧浓度：□□ %　　经皮脉搏氧饱和度：□□ %

用药60秒

心率：□□□次/分　　呼吸：□□次/分

吸入氧浓度：□□ %　　经皮脉搏氧饱和度：□□ %

用药90秒

心率：□□□次/分　　呼吸：□□次/分

吸入氧浓度：□□ %　　经皮脉搏氧饱和度：□□ %

研究医师（签名）：__________________

日期：201□年 □□月□□ 日□□时□□分

复核人（签名）：____________________

日期：201□年 □□月□□ 日□□时□□分

观察期记录

用药 120 秒

心率：□□次/分　　呼吸：□□次/分

吸入氧浓度：□□ %　　经皮脉搏氧饱和度：□□ %

用药 150 秒

心率：□□□次/分　　呼吸：□□次/分

吸入氧浓度：□□ %　　经皮脉搏氧饱和度：□□ %

用药 180 秒

心率：□□□次/分　　呼吸：□□次/分

吸入氧浓度：□□ %　　经皮脉搏氧饱和度：□□ %

研究医师（签名）：____________________

日期：201□ 年 □□月 □□ 日□□时 □□分

复核人（签名）：______________________

日期：201□ 年 □□月 □□ 日□□时 □□分

研究中心编号<br>□ □	受试者姓名缩写<br>□□□□	受试者编号<br>□□□□	观察期记录

用药结束后 5 分钟：

生命体征：

体温： □.□ ℃ **心率：**□□□ **次/分**

呼吸： □□**次/分** **血压：**□□ /□□ mmHg

经皮脉搏氧饱和度：□□ %

呼吸支持方式：

□NCPAP □BiPAP □NIPPV □sNIPPV □ nHFO

呼吸支持参数：

FiO2：□□%； PEEP □□ cmH_2O

（选填） PIP: □□cmH_2O **频率：**□ □**次/分或**□□ HZ

吸气时间 □.□**秒** **振幅：**□□cmH2O

研究医师（签名）：____________________

日期：201□ 年 □□月 □□ 日□□时 □□分

复核人（签名）：______________________

日期：201□ 年 □□月 □□ 日□□时 □□分

研究中心编号<br>□ □	受试者姓名缩写<br>□□□□	受试者编号<br>□□□□	观察期记录

观察期记录：出生后 72 小时

生后 72 小时内是否接受机械通气支持：□无 □ 有

(如有请填写以下内容)：

开始机械通气时间：201□ 年□□月□□ 日□□时□□ 分

开始机械通气原因：________________________________

呼吸机参数记录（初设稳定后）：

常频机械通气：是 □ 否 □

Fi02： □ □ %； PEEP □ □ cmH_2 PIP： □ □ cmH_2O

频率： □ □ 次/分 吸气时间□ . □ 秒

高频机械通气：是 □ 否 □

Fi02： %； MAP □ □cmH_2 振幅 □ □ cmH2O

频率： □ □ HZ 吸呼比 □：□

目前是否仍接受机械通气支持：□ 无 □ 有

研究医师（签名）：__________________

日期：201□ 年 □□月 □□ 日□□时 □□分

复核人（签名）：____________________

日期：201□ 年 □□月 □□ 日□□时 □□分

研究中心编号<br>□ □	受试者姓名缩写<br>□□□□	受试者编号<br>□□ □□	观察期记录

观察期记录：（出院日或死亡日）

是否正常出院：是 □ 否□

一般情况：

体重：□□□□ 克 身长：□ □ cm 头围 □ □ cm

生命体征：

体温： □ .□ ℃ 心率：□□ 次/分

呼吸： □ □次/分 血压：□ □ /□□ mmHg

经皮脉搏氧饱和度：□ □ %

住院期间呼吸支持小结

机械通气开始日期：201□ 年□□ 月□□ 日□□时□□分

机械通气结束日期：201□ 年□□ 月□□ 日□□时□□分

无创通气开始日期：201□ 年□□ 月□□ 日□□时□□分

无创通气结束日期：201□ 年□□ 月□□ 日□□时□□分

氧疗开始日期： 201□ 年□□ 月□□ 日□□时□□分

氧疗结束日期： 201□ 年□□ 月□□ 日□□时□□分

研究医师（签名）：________________

日期：201□ 年 □□月 □□ 日□□时 □□分

复核人（签名）：__________________

日期：201□ 年 □□月 □□ 日□□时 □□分

研究中心编号<br>□ □	受试者姓名缩写<br>□□□□	受试者编号<br>□□□□	观察期记录

观察期记录：（出院日或死亡日）

住院期间新生儿相关疾病发生情况：

1.大量肺出血：是□ 否□

发生日期：201□年□□月□□日□□时□□分 （如有）

临床转归：治愈□ 好转□ 未愈□ 死亡□（如有）

2. 气漏： 是□ 否□

发生日期：201□年□□月□□日□□时□□分 （如有）

临床转归：治愈□ 好转□ 未愈□ 死亡□（如有）

3.需要治疗的早产儿视网膜病： 是□ 否□

发生日期：201□年□□月□□日□□时□□分 （如有）

治疗方式：单抗注入□ 激光治疗□ 冷凝治疗□ （如有）

临床转归：治愈□ 好转□ 未愈□ 死亡□ （如有）

4. Ⅲ或Ⅳ度颅内出血： 是□ 否□

发生日期：201□年□□月□□日□□时□□分 （如有）

临床转归：治愈□ 好转□ 未愈□ 死亡□（如有）

研究医师（签名）：________________

日期：201□年□□月□□日□□时□□分

复核人（签名）：__________________

日期：201□年□□月□□日□□时□□分

研究中心编号<br>□ □	受试者姓名缩写<br>□□□□	受试者编号<br>□□ □□	观察期记录

观察期记录：（出院日或死亡日）

住院期间新生儿相关疾病发生情况（续）：

5.坏死性小肠结肠炎：是 □ 否 □

发生日期：201□年□□月□ □日□□时□□分 （如有）

临床转归：治愈 □ 好转 □ 未愈 □ 死亡 □（如有）

Bell 分级：Ia□ Ib□ Ⅱa □ Ⅱb □

治疗方式： 保守治疗 □ 手术治疗 □

3. hs-PDA： 是□ 否 □

发生日期：201 □年 □□月□ □日 □□时□□分 （如有）

治疗方式：药物治疗 □ 手术治疗 □ （如有）

临床转归：治愈 □ 好转 □ 未愈 □ 死亡 □（如有）

研究医师（签名）：________________

日期：201□ 年 □□月 □□ 日□□时 □□分

复核人（签名）：__________________

日期：201□ 年 □□月 □□ 日□□时 □□分

研究中心编号<br>□ □	受试者姓名缩写<br>□□□□	受试者编号<br>□□ □□	观察期记录

合并用药

无 □　　有□ 如有请填写下表

商品名或通用名	剂量/用法	使用原因	开始日期<br>（年/月/日）	结束日期<br>（年/月/日）	继续用药
			_____/__/__	_____/__/__	□
			_____/__/__	_____/__/__	□
			_____/__/__	_____/__/__	□
			_____/__/__	_____/__/__	□
			_____/__/__	_____/__/__	□
			_____/__/__	_____/__/__	□
			_____/__/__	_____/__/__	□
			_____/__/__	_____/__/__	□
			_____/__/__	_____/__/__	□
			_____/__/__	_____/__/__	□
			_____/__/__	_____/__/__	□
			_____/__/__	_____/__/__	□

研究医师（签名）：__________________

日期：201□ 年 □□月 □□ 日□□时 □□分

复核人（签名）：____________________

日期：201□ 年 □□月 □□ 日□□时 □□分

研究中心编号	受试者姓名缩写	受试者编号	观察期记录
□ □	□□□□	□□ □□	

不良事件记录表

（标准医学术语）记录所用观察到和直接询问得出的不良事件。每一栏记录一个不良事件。如果在研究期间有不良事件发生，请填写下表。无论有无不良反应，均应在此表下方签字<br>有无不良事件发生？有 □ 无 □				
不良事件名称				
开始发生日期	___年__月__日	___年__月__日	___年__月__日	___年__月__日
用药时间及剂量				
严重程度				
是否采取措施				
与本研究的关系				
所发生的不良事件的结局				
患者是否因此不良事件而退出研究				

研究医师（签名）：__________________

日期：201□ 年 □□月 □□ 日□□时 □□分

复核人（签名）：____________________

日期：201□ 年 □□月 □□ 日□□时 □□分

研究中心编号<br>□ □	受试者姓名缩写<br>□□□□	受试者编号<br>□□□□	观察期记录

研究完成情况总结

下列两项中，仅选择一项：

□ **受试者完成本项研究 （完成日期：** 201□年□□月□□日）

□ **受试者从本研究退出 （退出日期：** 201□年□□月□□日）

如果受试者退出研究，请在如下退出原因中选择一项：

退出原因（选择一个）：

□ 不良事件（请记录不良事件页）

□ **不符合入选/排除标准**

请注明：______________________________________

□ **违背方案**

请注明：______________________________________

□ **撤回知情同意书**

□ **其他**

请注明：______________________________________

研究医师（签名）：________________

日期：201□ 年 □□月 □□ 日□□时 □□分

复核人（签名）：__________________

日期：201□ 年 □□月 □□ 日□□时 □□分

研究中心编号<br>□ □	受试者姓名缩写<br>□□□□	受试者编号<br>□□□□	观察期记录

临床试验流程说明：

筛选期出生后：0-6 小时

不良事件观察期：外源性肺表面活性物质运用后 72 小时

访视点：

1.用药前 5 分钟，用药过程及用药后 5 分钟

2.生后 72 小时

3.校正胎龄 36 周或出院日或死亡日
